# Immunohistochemical Status Predicts Pathologic Complete Response to Neoadjuvant Therapy in HER2-Overexpressing Breast Cancers

**DOI:** 10.1245/s10434-024-16470-8

**Published:** 2024-11-09

**Authors:** Leah Winer, Karen J. Ruth, Richard J. Bleicher, Rajeswari Nagarathinam, Melissa McShane, Andrea S. Porpiglia, Mary T. Pronovost, Allison Aggon, Austin D. Williams

**Affiliations:** 1 Department of Surgical Oncology, Fox Chase Cancer Center, Philadelphia, PA USA; 2 Biostatistics Facility, Fox Chase Cancer Center, Philadelphia, PA USA; 3 Division of Breast Surgical Oncology, Department of Surgical Oncology, Fox Chase Cancer Center, Philadelphia, PA USA; 4 Department of Pathology, Fox Chase Cancer Center, Philadelphia, PA USA; 5 Department of Hematology/Oncology, Fox Chase Cancer Center, Philadelphia, PA USA

**Keywords:** Breast cancer, Human epidermal growth factor (HER2), Immunohistochemistry (IHC), In situ hybridization (ISH), Pathologic complete response (pCR)

## Abstract

**Background:**

Human epidermal growth factor receptor 2 (HER2) overexpression (HER2+) is defined by immunohistochemistry (IHC) and in situ hybridization (ISH) as IHC3+ or IHC2+/ISH+. Response differences to neoadjuvant anti-HER2 therapy (NT) in IHC3+ versus IHC2+/ISH+ breast cancer patients are poorly characterized. We explored whether pathologic complete response (pCR) varies by HER2 IHC status.

**Methods:**

Patients with stage I–III HER2+ breast cancer undergoing NT and surgery between 2013 and 2020 were identified from the National Cancer Database and stratified by IHC status. Breast and nodal pCR were analyzed.

**Results:**

Of 40,711 HER2+ patients, 83% were IHC3+ and 17% were IHC2+/ISH+. IHC3+ patients were more likely to be hormone receptor (HR)-negative (33 vs. 21%), have cT3/4 tumors (24 vs. 21%), and be cN+ (52 vs. 47%; all *p *< 0.0001). Breast conservation rates were similar (each 43%, *p *= 0.32), although IHC3+ axillary lymph node dissection rates were lower (41 vs. 45%, *p *< 0.0001). Among all patients, breast pCR was 49%, while nodal pCR was 64%. Compared with IHC2+/ISH+, IHC3+ had higher unadjusted breast (54 vs. 22%, *p *< 0.0001) and nodal (69 vs. 37%, *p* < 0.0001) pCR rates. When stratified by HR status, pCR was lower for HR+ disease but remained higher among IHC3+ patients. Analysis of T1cN0 primaries mirrored these trends. In multivariable analysis, IHC3+ remained an independent predictor of breast (odds ratio [OR] 3.91, confidence interval [CI] 3.65–4.19, *p* < 0.0001) and nodal (OR 3.40, CI 3.12–3.71, *p* < 0.0001) pCR.

**Conclusion:**

HER2 IHC status predicts pCR and may help select breast cancer patients who derive the greatest benefit from NT. These findings provide further evidence that revision of HER2 classification may improve clinical management.

**Supplementary Information:**

The online version contains supplementary material available at 10.1245/s10434-024-16470-8.

Human epidermal growth factor receptor 2 (HER2) is a membrane tyrosine kinase receptor overexpressed in approximately 15% of invasive breast cancers.^1^ According to the National Comprehensive Cancer Network (NCCN) clinical oncology guidelines, all patients with newly diagnosed invasive breast cancer should undergo HER2 testing with immunohistochemistry (IHC) to evaluate protein expression levels.^2^ For those with equivocal results (IHC2+), reflex in situ hybridization (ISH) is required to quantify HER2 gene expression. By convention, HER2 overexpression (HER2+) is defined as either IHC3+ or IHC2+/ISH+, with previous studies demonstrating high concordance between IHC expression and ISH amplification assays.^3–5^

For more than two decades, clinicians have utilized HER2 status to prognosticate disease and select patients for clinical trial enrollment and targeted regimens. However, the current system of HER2 classification may underappreciate clinically relevant differences in tumor biology. A recent paradigm-shifting clinical trial identified the efficacy of trastuzumab deruxtecan in IHC1+ or IHC2+/ISH− patients with metastatic breast cancer, creating the new category of ‘HER2-low’ disease.^6^ Even among HER2+ patients undergoing neoadjuvant (NT) anti-HER2 therapy, which has become standard of care in many cases, breast pathologic complete response (pCR) rates approach only 50%, indicating variable treatment potential.^7–10^ Thus, it remains unclear whether stratifying by IHC status is clinically relevant in the management of HER2+ patients.

To date, few studies have evaluated clinical or histologic differences between IHC3+ and IHC2+/ISH+ invasive breast cancer patients treated with NT-targeted therapy. In one single-institution study, Horimoto et al. detected a pCR rate of 45% versus 21% in IHC3+ and IHC2+/ISH+ patients, but this finding was not statistically significant. Similarly, the authors observed no differences in histologic characteristics or disease-free or overall survival, but the study was limited by its small sample size (*N* = 134).^11^ In a somewhat larger retrospective cohort study, Atallah et al.^12^ found that pCR rates were significantly better among IHC3+ versus IHC2+/ISH+ breast cancer patients, suggesting that HER2 protein overexpression may predict response to anti-HER2 therapy. The objective of this study was to use a large population database to explore whether clinicopathologic variables and pCR rates vary based on HER2 IHC status among patients with HER2+ breast cancer undergoing NT anti-HER2 therapy followed by surgery.

## Methods

### Patient Cohort and Study Variables

**Fig. 1:**
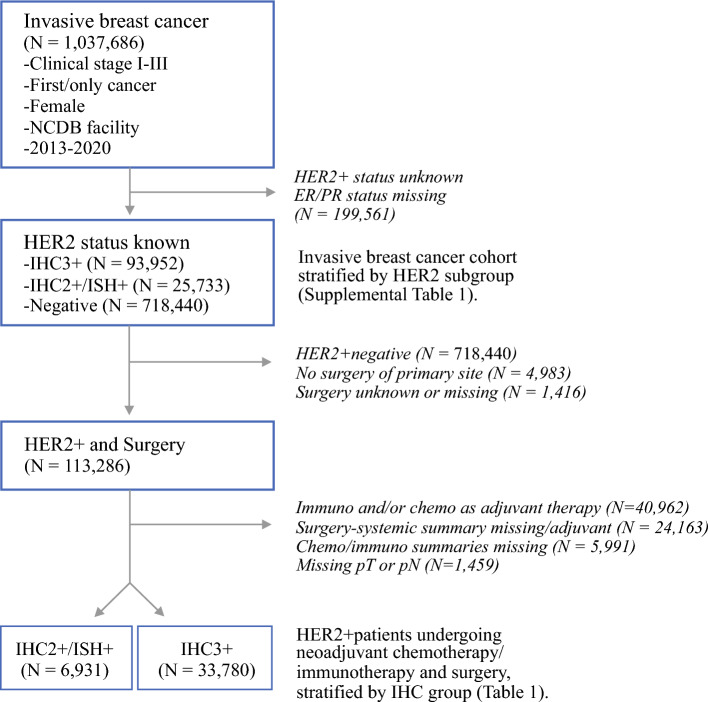
STROBE diagram illustrating the inclusion and exclusion criteria for NCDB breast cancer patients. *STROBE* strengthening the reporting of observation studies in epidemiology, *NCDB* National Cancer Database, *HER2* human epidermal growth factor receptor 2, *ER* estrogen receptor, *PR* progesterone receptor, *IHC* immunohistochemistry, *ISH* in situ hybridization

Breast cancer patient data were collected from the American College of Surgeons’ (ACS) National Cancer Database (NCDB) participant user file (PUF). The NCDB is a joint project of the ACS’ Commission on Cancer and the American Cancer Society that includes de-identified patient registry data from more than 1500 nationally accredited cancer programs that capture 70% of all malignancies in the United States (US).^13^ The NCDB PUF was queried for all female patients with invasive breast cancer, clinical stages I–III, treated between 2013 and 2020 (*N *= 1,037,686) with known HER2 and hormone receptor (HR) status (*n* = 838,125) (Fig. 1). The final cohort included HER2+ breast cancer patients who underwent NT systemic treatment, followed by surgery (*n* = 40,711). NT was determined using the NCDB’s systemic therapy sequence variable, as well as by taking the time from diagnosis to the receipt of systemic therapies compared with the time from diagnosis to surgery. Those with missing HER2 status, surgical history, or systemic treatment data, as well as patients not undergoing NT, were excluded. Because the NCDB only coded uniformly for targeted therapy in 2013, patients who received treatment prior to this year were excluded. Per the same 2013 PUF update, trastuzumab and pertuzumab were recategorized as immunotherapy, rather than as chemotherapy, under the NCDB’s systemic treatment category. Therefore, HER2+ patients who underwent both chemotherapy and immunotherapy in the NT setting were presumed to have received standard-of-care anti-HER2 treatment.

HER2+ patients were then grouped by IHC status: IHC0, IHC1+, and ICH2+/ISH− were defined as HER2-negative, and IHC2+/ISH+ and IHC3+ were defined as HER2 overexpressing. For HER2 IHC2+/ISH+ patients, ISH results are expressed as a dual-probe HER2/CEP17 ratio or single-probe HER2 count. For dual-probe results, HER2/CEP17 ≥ 2 was considered ISH+ per College of American Pathologists’ guidelines.^14^

Among HER2+ patients who underwent NT and surgery, the primary outcomes were breast pCR, nodal pCR, and total pCR (both breast and axilla), defined as the absence of residual invasive disease in the resected specimen and/or sampled lymph nodes.^15^ Breast pCR was determined by comparing American Joint Committee on Cancer (AJCC) clinical and pathologic T categories. For patients diagnosed after 2017, ypT was available in the NCDB; for the remaining patients, pT was used despite not being specified as ypT category. If pT was unknown, then pathologic tumor size was assessed, if available, and categorized by AJCC staging guidelines. Patients noted to be yp or pT0/Tis were considered to have a pCR. Patients with a decreased pT relative to cT, but not pCR, were characterized as having partial response, and those with pT equal to or greater than cT were considered to have no response. Similarly, nodal pCR was evaluated in clinically node-positive patients (cN+). If either pN or ypN was unknown, the number of regional positive lymph nodes was assessed for pN per AJCC staging guidelines. Patients who became pN0 were considered to have a pCR; patients were coded as partial response if pN was lower than cN, and as no response otherwise. Among cN+ patients, total pCR was defined as both breast and nodal pCR (ypT0/Tis ypN0).

### Statistical Analysis

The primary objective was to determine whether pCR differed by HER2+ IHC subgroup. Demographic and clinicopathologic characteristics were compared between IHC3+ and IHC2+/ISH+ patients using Chi-square tests; for age at diagnosis, medians and 25th and 75th percentiles for interquartile range (IQR) are shown and compared using Kruskal–Wallis tests. For breast, nodal, and total pCR, the proportions of patients with complete, partial, and no pathologic responses were compared by HER2+ subgroup and HR status (estrogen receptor-positive [ER+] or progesterone receptor-positive [PR+] vs. ER-negative [ER−] and PR-negative [PR−]) using Chi-square tests. For both nodal and total pathologic response outcomes, cN0 patients were excluded. The association of HER2+ IHC status with breast, nodal, and total pCR were examined separately using multivariable logistic regression. To account for within-facility correlation, generalized estimating equations with robust standard errors were utilized, adjusting for demographic factors (i.e., age [continuous], race [White, African American, Asian, other/unknown], Hispanic ethnicity [yes, no, unknown], Charlson–Deyo Comorbidity score [0, 1, 2+]), socioeconomic factors (i.e., type of insurance and income by zip code-based quartiles), and tumor-related factors, including histology, HR status, grade, clinical T and N categories, and type of surgery. Effect sizes are presented as odds ratios (ORs) for the probability of pCR with 95% confidence limits for covariate-adjusted models. In separate analyses, we included facility type (academic/research, comprehensive community, and community program) in the models after excluding patients age < 40 years, for whom the NCDB does not provide these data.

The HER2 IHC2+/ISH+ subgroup was determined by ISH assay as previously described. In an ad hoc subset analysis of IHC2+/ISH+ patients, we examined a dose-response relationship between the HER2/CEP17 ratio and breast, nodal, and total pCR using separate models: (1) dichotomous variable <2 vs. ≥2; and (2) categorical variable with seven levels (0.20–1.99, 2.00–2.25, 2.26–2.50, 2.51–3.00, 3.01–4.00, 4.01–6.00, and 6.01–96.00), with increasing intervals similar to logarithmic increases. Results are presented as unadjusted pCR proportions and, for the latter model, covariate-adjusted ORs. All analyses were performed using SAS version 9.4 (SAS Institute, Inc., Cary, NC, USA), using two-sided tests, and significance was set at *p* < 0.05. The study was deemed exempt by the Fox Chase Cancer Center Institutional Review Board.

## Results

### Immunohistochemistry (IHC) 3+ Versus IHC2+/In Situ Hybridization (ISH)+ Demographics

This study included a total of 40,711 patients, of whom 83.0% were IHC3+ (*n = *33,780) and 17.0% were IHC2+/ISH+ (*n = *6931). Clinical and demographic data for HER2+ patients grouped by IHC status are provided in Table 1. Compared with IHC2+/ISH+ patients, IHC3+ patients were younger (53 vs. 55 years, *p* < 0.0001) with better performance status (Charlson–Deyo 0: 87.1% vs. 84.6%, *p* < 0.0001). IHC3+ patients were more likely to have ductal histology (96.0% vs. 94.3%) and high-grade tumors (57.9% vs. 53.4%) with lower rates of lymphovascular invasion (15.6% vs. 22.3%) and HR expression (66.9% vs. 79.3%, all *p* < 0.0001). IHC3+ patients tended to present with cT3/4 disease (23.7% vs. 21.0%,* p* < 0.0001), but breast conservation rates were comparable between groups (42.8% vs. 43.4%, *p* = 0.32). Although IHC3+ patients were also more likely to present with clinically detectable nodal disease (cN+ 52.2% vs. 46.8%, *p* < 0.0001), rates of axillary lymph node dissection were lower among this cohort (41.1% vs. 44.8%, *p* < 0.0001). Demographics for all patients with complete data for HER2 and HR status (*n = *838,125), including comparisons with HER2-negative patients, are shown in electronic supplementary material (ESM) Table 1.

**Table 1:** Demographics and clinicopathologic characteristics of HER2+ patients who underwent neoadjuvant chemotherapy and surgery stratified by IHC status

	Overall	HER2 status		*p*-Value
		*IHC2+/ISH+*	*IHC3+*	
*N*	40,711	6931 (17.0%)	33,780 (83.0%)	
Age at diagnosis, years [median (IQR)]	53 (44–62)	55 (46–64)	53 (44–61)	< 0.0001
*Race/ethnicity*				
White	31,184 (76.6)	5261 (75.9)	25,923 (76.7)	< 0.0001
Black	5689 (14.0)	1074 (15.5)	4615 (13.7)	
Asian	2692 (6.6)	408 (5.9)	2284 (6.8)	
Other/unknown	1146 (2.8)	188 (2.7)	958 (2.8)	
*Charlson–Deyo score*				
0	35,279 (86.7)	5864 (84.6)	29,415 (87.1)	< 0.0001
1	4232 (10.4)	815 (11.8)	3417 (10.1)	
≥2	1200 (2.9)	252 (3.6)	948 (2.8)	
*Insurance status*				
Not insured	1188 (2.9)	183 (2.6)	1005 (3.0)	< 0.0001
Private insurance	26,535 (65.2)	4326 (62.4)	22,209 (65.7)	
Medicaid	4450 (10.9)	709 (10.2)	3741 (11.1)	
Medicare	7590 (18.6)	1552 (22.4)	6038 (17.9)	
Other government	549 (1.3)	80 (1.2)	469 (1.4)	
Unknown	399 (1.0)	81 (1.2)	318 (0.9)	
*No high-school degree*				
≥17.6%	6782 (16.7)	1104 (15.9)	5678 (16.8)	0.06
10.9–17.5%	8697 (21.4)	1444 (20.8)	7253 (21.5)	
6.3–10.8%	9901 (24.3)	1682 (24.3)	8219 (24.3)	
<6.3%	9293 (22.8)	1610 (23.2)	7683 (22.7)	
Unknown	6038 (14.8)	1091 (15.7)	4947 (14.6)	
*Median income*				
<$40,227	5064 (12.4)	904 (13.0)	4160 (12.3)	0.002
$40,227–$50,353	6706 (16.5)	1127 (16.3)	5579 (16.5)	
$50,354–$63,332	8135 (20.0)	1278 (18.4)	6857 (20.3)	
≥$63,333	14,702 (36.1)	2519 (36.3)	12,183 (36.1)	
Unknown	6104 (15.0)	1103 (15.9)	5001 (14.8)	
*Institution type*^*a*^				
Community Cancer Center	1928 (4.7)	324 (4.7)	1604 (4.7)	< 0.0001
Comprehensive Community Cancer Program	13,306 (32.7)	2317 (33.4)	10,989 (32.5)	
Academic/Research Program	11,700 (28.7)	2110 (30.4)	9590 (28.4)	
Integrated Network Cancer Program	7547 (18.5)	1341 (19.3)	6206 (18.4)	
*Histology*				
Ductal	38,956 (95.7)	6536 (94.3)	32,420 (96.0)	< 0.0001
Lobular	1199 (2.9)	287 (4.1)	912 (2.7)	
Mixed/other	556 (1.4)	108 (1.6)	448 (1.3)	
*Estrogen receptor*				
Negative	13,728 (33.7)	1563 (22.6)	12,165 (36.0)	< 0.0001
Positive	26,983 (66.3)	5368 (77.4)	21,615 (64.0)	
*Progesterone receptor*				
Negative	19,657 (48.3)	2345 (33.8)	17,312 (51.2)	< 0.0001
Positive	21,054 (51.7)	4586 (66.2)	16,468 (48.8)	
*ER/PR*				
ER/PR−	12,617 (31.0)	1433 (20.7)	11,184 (33.1)	< 0.0001
ER/PR+	28,094 (69.0)	5498 (79.3)	22,596 (66.9)	
*Tumor grade*				
Low	1093 (2.7)	273 (3.9)	820 (2.4)	< 0.0001
Intermediate	14,661 (36.0)	2721 (39.3)	11,940 (35.3)	
High	23,254 (57.1)	3703 (53.4)	19,551 (57.9)	
Undifferentiated	38 (0.1)	11 (0.2)	27 (0.1)	
Unknown or missing	1665 (4.1)	223 (3.2)	1442 (4.3)	
*Lymphovascular invasion*				
Absent	21,814 (53.6)	3616 (52.2)	18,198 (53.9)	< 0.0001
Present	6797 (16.7)	1543 (22.3)	5254 (15.6)	
Unknown	12,079 (29.7)	1770 (25.5)	10,309 (30.5)	
*Clinical tumor stage*				
cT1	8685 (21.3)	1482 (21.4)	7203 (21.3)	< 0.0001
cT2	22,579 (55.5)	3994 (57.6)	18,585 (55.0)	
cT3	6365 (15.6)	1010 (14.6)	5355 (15.9)	
cT4	3082 (7.6)	445 (6.4)	2637 (7.8)	
*Clinical nodal stage*				
cN0	19,847 (48.8)	3686 (53.2)	16,161 (47.8)	< 0.0001
cN1	17,212 (42.3)	2696 (38.9)	14,516 (43.0)	
cN2	2019 (5.0)	309 (4.5)	1710 (5.1)	
cN3	1633 (4.0)	240 (3.5)	1393 (4.1)	
*Breast surgery*				
Partial mastectomy	17,450 (42.9)	3008 (43.4)	14,442 (42.8)	0.32
Mastectomy	23,261 (57.1)	3923 (56.6)	19,338 (57.2)	
*Axillary surgery*				
None	552 (1.4)	77 (1.1)	475 (1.4)	< 0.0001
SLNB alone	23,080 (56.7)	3739 (53.9)	19,341 (57.3)	
SNLB then ALND	7083 (17.4)	1471 (21.2)	5612 (16.6)	
ALND alone	9914 (24.4)	1635 (23.6)	8279 (24.5)	
Unknown	82 (0.2)	9 (0.1)	73 (0.2)	

Data are expressed as *n* (%) unless otherwise specified

*ALND* axillary lymph node dissection, *ER* estrogen receptor, *HER2* human epidermal growth factor receptor 2, *IHC* immunohistochemistry, *ISH* in situ hybridization, *IQR* interquartile range, *PR* progesterone receptor, *SLNB* sentinel lymph node biopsy

^a^Facility type excludes ages 18–39 years, where these data are suppressed

### Unadjusted Breast and Nodal Pathologic Complete Response (pCR) Analyses

Pathologic response rates were reported separately for the breast and axillary lymph nodes, as well as combined (total pCR) for cN+ patients (Table 2). For the whole cohort, breast pCR was 48.8% (*n = *19,874). Among the *n = *20,864 patients with cN+ disease, nodal pCR was 63.7% (*n = *13,299) and total pCR was 44.3% (*n = *9233). Stratification by IHC status revealed dramatically improved unadjusted pCR rates in IHC3+ versus IHC2+/ISH+ patients in the breast (54.3% vs. 22.0%, *p* < 0.0001), axillary lymph nodes (68.7% vs. 37.0%,* p* < 0.0001), and combined breast and axilla (49.4% vs. 16.3%, *p* < 0.0001).

**Table 2:** Breast, nodal, and total pathologic response to neoadjuvant therapy among HER2+ patients stratified by **A** IHC and HR status and **B** breast pathologic response rates among the T1cN0 cohort stratified by IHC and HR status

A												
Overall						HR+				HR−		
	Overall	HER2 status		*p*-Value	Overall	HER2 status		*p*-Value	Overall	HER2 status		*p*-Value
		*IHC2+/ISH+*	*IHC3+*			*IHC2+/ISH+*	*IHC3+*			*IHC2+/ISH+*	*IHC3+*	
*N* (row %)	40,711	7144 (17.0)	35,026 (87.0)	<0.0001	28,094	5498 (19.6)	22,596 (80.4)	<0.0001	12,617	1433 (11.4)	11,184 (88.6)	<0.0001
*Breast response [N (col%)]*												
None	9065 (22.3)	2445 (35.3)	6620 (19.6)		7176	2078 (37.8)	5098 (22.6)		1889	367 (25.6)	1522 (13.6)	
Partial	11,772 (28.9)	2961 (42.7)	88,811 (26.1)		9264	2464 (44.8)	6800 (30.1)		2508	497 (34.7)	2011 (18.0)	
Complete	19,874 (48.8)	1525 (22.0)	18,349 (54.3)		11,654	956 (17.4)	10,698 (47.3)		8220	569 (39.7)	7651 (68.4)	
*N*^a^ (row %)	20,864	3245 (15.6)	17,619 (84.4)	<0.0001	13,859	2568 (18.5)	11,291 (81.5)	<0.0001	7005	677 (9.7)	6328 (90.3)	<0.0001
*Nodal response [n (col%)]*												
None	6664 (31.9)	1834 (56.5)	4830 (27.4)		5143	1574 (61.3)	3569 (31.6)		1521	260 (38.4)	1261 (19.9)	
Partial	901 (4.3)	211 (6.5)	690 (3.9)		620	166 (6.5)	454 (4.0)		281	45 (6.6)	236 (3.7)	
Complete	13,299 (63.7)	1200 (37.0)	12,099 (68.7)		8096	828 (32.2)	7268 (64.4)		5203	372 (54.9)	4831 (76.3)	
*Breast + nodal response [n (col%)]*												
None or one pCR	11,631 (55.7)	2716 (83.7)	8915 (50.6)	<0.0001	8684	2249 (87.6)	6453 (57.0)	<0.0001	2947	467 (69.0)	2480 (39.2)	<0.0001
Both pCR	9233 (44.3)	529 (16.3)	8704 (49.4)		5175	319 (12.4)	4856 (43.0)		4058	210 (31.0)	3848 (60.8)	

B												
Overall						HR+				HR-		
	Overall	HER2 status		*p*-Value	Overall	HER2 status		*p*-Value	Overall	HER2 status		*p*-Value
		*IHC2+/ISH+*	*IHC3+*			*IHC2+/ISH+*	*IHC3+*			*IHC2+/ISH+*	*IHC3+*	
*N* (row%)	3993	759 (19.0)	3234 (81.0)	<0.0001	2997	630 (21.0)	2367 (79.0)	<0.0001	996	129 (12.9)	867 (87.1)	<0.0001
*Breast response [n (col%)]*												
None	2147 (53.8)	594 (78.3)	1681 (52.0)		1834	518 (82.2)	1316 (55.6)		313	76 (58.9)	237 (27.3)	
Complete	1846 (46.2)	165 (21.7)	1553 (48.0)		1163	112 (17.8)	1051 (44.4)		683	53 (41.1)	630 (72.7)	

*HER2* human epidermal growth factor receptor 2, *HR* hormone receptor, *IHC* immunohistochemistry, *ISH* in situ hybridization, *pCR* pathological complete response

Next, pCR was examined among clinically relevant subgroups, including HR-expressing and T1c tumors, for which the benefit of NT anti-HER2 is less clear (Table 2). After stratifying by HR status, breast and nodal pCR rates were superior among all HR-negative subgroups; however, IHC3+ pCR rates still exceeded those of IHC2+/ISH+ tumors, regardless of HR status HR+ breast pCR: 47.3% vs. 17.4%, and nodal pCR: 64.4% vs. 32.2%, *p* < 0.0001; HR− breast pCR: 68.4% vs. 39.7%, and nodal pCR: 76.3% vs. 54.9%, *p* < 0.0001). Among T1cN0 patients (*n = *3993), 46.2% (*n = *1846) had a breast pCR, with significantly higher pCR rates among IHC3+ patients (48.0% vs. 21.7%, *p* < 0.0001). Similar to the whole cohort, pCR was lower among HR+ T1c patients; despite HR status, breast pCR was improved among IHC3+ versus IHC2+/ISH+ (44.4% vs. 17.8%, *p* < 0.0001).

### Adjusted pCR Analyses

Multivariable analyses were performed to adjust for socioeconomic, clinical, and pathologic factors associated with breast, nodal, and total pCR (Tables 3, 4 and 5). For all three outcomes, IHC3+ status conferred a significantly increased odds of pCR (breast: odds ratio [OR] 3.91, confidence interval [CI] 3.65–4.19, *p* < 0.0001; nodal: OR 3.40, CI 3.12–3.71, *p* < 0.0001; total: OR 4.58, CI 4.10–5.11, *p* < 0.0001). Additional covariates associated with breast pCR included higher median income, tumor grade, and clinical nodal category (all *p* < 0.05). Variables significantly associated with nodal pCR included private insurance status, non-ductal histology, and higher tumor grade (all *p* < 0.05). Similar to previous subgroup analyses, HR expression was associated with an approximately twofold increased probability of residual disease in both the breast and axilla (breast pCR: OR 0.41, CI 0.39–0.43, *p* < 0.0001; nodal pCR: OR 0.53, CI 0.50–0.57, *p* < 0.0001). Mastectomy was also associated with non-pCR (breast pCR: OR 0.85, CI 0.81–0.89, *p* < 0.0001; nodal pCR: OR 0.79, CI 0.74–0.84, *p* < 0.0001)

**Table 3:** Multivariable analysis of variables associated with achieving breast pathologic complete response

	Multivariable		
	OR	95% CI	*p*-Value
Age at diagnosis (continuous), 5-year difference	0.96	0.95–0.97	<0.0001
*Race/ethnicity*			
White (reference)	–	–	0.44
Black	0.97	0.90–1.03	
Asian	1.01	0.93–1.09	
Other/unknown	1.08	0.95–1.23	
*Hispanic*			
No (reference)	–	–	0.004
Yes	1.07	0.99–1.15	
Unknown	0.76	0.63–0.92	
*Charlson–Deyo score*			
0 (reference)	–	–	<0.0001
1	0.94	0.87–1.00	
≥2	0.74	0.66–0.84	
*Insurance status*			
Medicaid (reference)	–	–	0.004
Medicare	0.94	0.86–1.03	
Private insurance	1.06	0.99–1.14	
Other	1.03	0.91–1.17	
*Median income*			
<$40,227 (reference)	–	–	0.004
$40,227–$50,353	1.09	1.01–1.18	
$50,354–$63,332	1.16	1.07–1.25	
≥$63,333	1.12	1.05–1.20	
Unknown	1.14	1.05–1.23	
*Histology*			
Ductal (reference)	–	–	0.0003
Lobular	0.77	0.68–0.88	
Other	1.13	0.92–1.38	
*Hormone receptor*			
Negative (reference)	–	–	<0.0001
Positive (ER+ and/or PR+)	0.41	0.39–0.43	
*Tumor grade*			
Low (reference)	–	–	<0.0001
Intermediate	1.72	1.49–1.99	
High	2.23	1.93–2.58	
Undifferentiated	1.16	0.53–2.26	
Unknown	2.22	1.85–2.66	
*Clinical tumor stage*			
cT1 (reference)	–	–	<0.0001
cT2	0.93	0.88–0.98	
cT3	0.83	0.78–0.90	
cT4	0.79	0.72–0.87	
*Clinical nodal stage*			
cN0 (reference)	–	–	0.005
cN1	1.04	0.99–1.09	
cN2	1.16	1.04–1.28	
cN3	1.16	1.04–1.29	
*Breast surgery*			
Partial mastectomy (reference)	–	–	<0.0001
Mastectomy	0.85	0.81–0.89	
*HER2 histology*			
IHC2+/ISH+ (reference)	–	–	<0.0001
IHC3+	3.91	3.65–4.19	

*CI* confidence interval, *ER* estrogen receptor, *HER2* human epidermal growth factor receptor 2, *IHC* immunohistochemistry, *ISH* in situ hybridization, *OR* odds ratio, *PR* progesterone receptor

**Table 4:** Multivariable analysis of variables associated with achieving axillary lymph node pathologic complete response

	Multivariable		
	OR	95% CI	*p*-Value
Age at diagnosis (continuous), 5-year difference	0.96	0.94–0.97	< 0.0001
*Race/ethnicity*			
White (reference)	–	–	0.002
Black	0.88	0.81–0.96	
Asian	1.08	0.96–1.22	
Other/unknown	1.14	0.91–1.33	
*Hispanic*			
No (reference)	–	–	0.07
Yes	1.10	0.98–1.22	
Unknown	0.83	0.66–1.05	
*Charlson–Deyo score*			
0 (reference)	–	–	0.007
1	0.90	0.82–0.99	
≥2	0.78	0.66–0.93	
*Insurance status*			
Medicaid (reference)	–	–	0.009
Medicare	1.01	0.89–1.14	
Private insurance	1.13	1.03–1.24	
Other	1.00	0.85–1.17	
Median income			
<$40,227 (reference)	–	–	0.76
$40,227–$50,353	0.99	0.89–1.10	
$50,354–$63,332	0.99	0.88–1.10	
≥$63,333	1.04	0.95–1.15	
Unknown	1.00	0.89–1.12	
*Histology*			
Ductal (reference)	–	–	0.007
Lobular	1.28	1.06–1.53	
Mixed/other	1.27	1.00–1.62	
Hormone receptor			
Negative (reference)	–	–	< 0.0001
Positive (ER+ and/or PR+)	0.53	0.50–0.57	
*Tumor grade*			
Low (reference)	–	–	< 0.0001
Intermediate	1.50	1.21–1.86	
High	1.80	1.45–2.24	
Undifferentiated	1.20	0.51–2.82	
Unknown	1.79	1.40–2.29	
*Clinical tumor stage*			
cT1 (reference)	–	–	0.21
cT2	1.03	0.95–1.13	
cT3	0.96	0.87–1.05	
cT4	0.96	0.85–1.07	
*Clinical nodal stage*			
cN1 (reference)	–	–	0.04
cN2	0.87	0.78–0.97	
cN3	1.01	0.90–1.13	
*Breast surgery*			
Partial mastectomy (reference)	–	–	< 0.0001
Mastectomy	0.79	0.74–0.84	
HER2 histology			
IHC2+/ISH+ (reference)	–	–	< 0.0001
IHC3+	3.40	3.12–3.71	

*CI* confidence interval, *ER* estrogen receptor, *HER2* human epidermal growth factor receptor 2, *IHC* immunohistochemistry, *ISH* in situ hybridization, *OR* odds ratio, *PR* progesterone receptor

**Table 5:** Multivariable analysis of variables associated with achieving both breast and nodal pCR (i.e. total pCR)

	Multivariable		
	OR	95% CI	*p*-Value
Age at diagnosis (continuous), 5-year difference	0.96	0.95–0.98	< 0.0001
*Race/ethnicity*			
White (reference)	–	–	0.15
Black	0.91	0.83–0.99	
Asian	0.98	0.88–1.10	
Other/unknown	1.07	0.90–1.28	
*Hispanic*			
No (reference)	–	–	0.03
Yes	1.09	0.99–1.22	
Unknown	0.80	0.63–1.01	
*Charlson–Deyo score*			
0 (reference)	–	–	0.001
1	0.91	0.83–0.99	
≥2	0.75	0.63–0.89	
*Insurance status*			
Medicaid (reference)	–	–	0.001
Medicare	0.93	0.82–1.07	
Private insurance	1.11	1.01–1.22	
Other	0.98	0.83–1.15	
Median income			
<$40,227 (reference)	–	–	0.50
$40,227–$50,353	0.98	0.89–1.08	
$50,354–$63,332	1.06	0.96–1.18	
≥$63,333	1.05	0.95–1.15	
Unknown	1.05	0.94–1.17	
*Histology*			
Ductal (reference)	–	–	0.55
Lobular	0.94	0.78–1.14	
Mixed/other	1.12	0.89–1.40	
Hormone receptor			
Negative (reference)	–	–	<0.0001
Positive (ER+ and/or PR+)	0.46	0.44–0.49	
*Tumor grade*			
Low (reference)	–	–	< 0.0001
Intermediate	1.84	1.42–2.38	
High	2.12	1.64–2.75	
Undifferentiated	0.99	0.33–2.96	
Unknown	2.16	1.63–2.87	
*Clinical tumor stage*			
cT1 (reference)	–	–	<0.0001
cT2	0.82	0.75–0.89	
cT3	0.72	0.65–0.80	
cT4	0.74	0.66–0.83	
*Clinical nodal stage*			
cN1 (reference)	–	–	0.11
cN2	1.05	0.95–1.17	
cN3	1.13	1.01–1.26	
*Breast surgery*			
Partial mastectomy (reference)	–	–	< 0.0001
Mastectomy	0.83	0.77–0.88	
*HER2 histology*			
IHC2+/ISH+ (reference)	–	–	< 0.0001
IHC3+	4.58	4.10–5.11	

*CI* confidence interval, *ER* estrogen receptor, *HER2* human epidermal growth factor receptor 2, *IHC* immunohistochemistry, *ISH* in situ hybridization, *OR* odds ratio, *pCR* pathologic complete response, *PR* progesterone receptor

Among the 44.3% of patients who achieved pCR in both the breast and axilla, covariates associated with total pCR included private insurance and tumor grade, while factors associated with higher odds of residual disease included older age, higher Charlson–Deyo score, HR expression, greater cT category, and mastectomy. Lobular histology was not a significant predictor of total pCR, with differing effects in each breast (OR 0.77, CI 0.68–0.88, *p* < 0.05) and nodal response (OR 1.27, CI 1.06–1.53, *p* < 0.05).

### IHC2+/ISH+ Adjusted Subanalyses

Given the greater frequency of breast, nodal, and total pCR among HER2 IHC3+ patients, as well as the finding that IHC3+ status is a strong independent predictor of pCR, additional analyses were performed among the HER2 IHC2+/ISH+ cohort. First, a subset analysis was performed to examine the relationship between the HER2/CEP17 ratio and breast, nodal, and total pCR (ESM Table 2). After demonstrating a direct relationship between the HER2/CEP17 ratio and therapeutic response, multivariable analysis was performed to identify covariates associated with total pCR among IHC2+/ISH+ patients (Table 6). This revealed that tumor grade, median income, and higher HER2/CEP17 ratio, an indicator of HER2+ gene expression, were predictive of pCR among HER2 IHC2+/ISH+ patients. A pCR was significantly less probable among HER2 IHC2+/ISH+ patients with HR+ disease or who underwent mastectomy.

**Table 6:** Multivariable analysis of factors associated with HER2+ IHC2+/ISH+ patients achieving both breast and nodal pCR (i.e. total pCR)

	Multivariable		
	OR	95% CI	*p*-Value
Age at diagnosis (continuous), 5-year difference	0.97	0.91–1.03	0.30
*Race/ethnicity*			
White (reference)	–	–	0.77
Black	0.98	0.71–1.33	
Asian	1.20	0.74–1.94	
Other/unknown	1.28	0.67–2.44	
*Hispanic*			
No (reference)	–	–	0.63
Yes	1.21	0.79–1.85	
Unknown	1.28	0.67–2.44	
*Charlson–Deyo score*			
0 (reference)	–	–	0.72
1	0.87	0.60–1.25	
≥2	1.04	0.56–1.93	
*Insurance status*			
Medicaid (reference)	–	–	0.19
Medicare	0.97	0.59–1.60	
Private insurance	1.20	0.82–1.75	
Other	0.73	0.40–1.35	
*Median income*			
<$40,227 (reference)	–	–	0.008
$40,227–$50,353	1.22	0.80–1.88	
$50,354–$63,332	2.02	1.35–3.03	
≥$63,333	1.35	0.91–1.99	
Unknown	1.31	0.79–2.31	
*Histology*			
Ductal (reference)	–	–	0.04
Lobular	2.32	1.38–3.91	
Mixed/other	1.31	0.64–2.70	
*Hormone receptor*			
Negative (reference)	–	–	<0.0001
Positive (ER+ and/or PR+)	0.35	0.27–0.44	
*Tumor grade*			
Low (reference)	–	–	<0.0001
Intermediate	1.86	1.42–2.39	
High	3.66	1.64–2.75	
Undifferentiated	20.68	1.45–294.97	
Other	4.09	1.40–11.91	
*Clinical tumor stage*			
cT1 (reference)	–	–	0.13
cT2	0.74	0.55–1.01	
cT3	0.64	0.45–0.92	
cT4	0.67	0.42–1.07	
*Clinical nodal stage*			
cN1 (reference)	–	–	0.81
cN2	1.15	0.77–1.71	
cN3	1.01	0.67–1.52	
*Breast surgery*			
Partial mastectomy (reference)	–	–	0.01
Mastectomy	0.72	0.56–0.92	
HER2/CEP ratio			
0.20–1.99	1.18	0.76–1.84	<0.0001
2.00–2.25 (reference)	–	–	
2.26–2.50	1.14	0.77–1.70	
2.51–3.00	1.38	0.93–2.04	
3.01–4.00	1.76	1.20–2.59	
4.01–6.00	2.75	1.85–4.09	
≥6.01	3.45	2.26–5.28	

*CEP17* centromere enumerator probe 17, *CI* confidence interval, *ER* estrogen receptor, *HER2* human epidermal growth factor receptor 2, *IHC* immunohistochemistry, *ISH* in situ hybridization, *OR* odds ratio, *pCR* pathologic complete response, *PR* progesterone receptor

## Discussion

The membrane tyrosine kinase receptor HER2 is implicated in breast cancer cell growth, differentiation, and angiogenesis, leading to aggressive disease biology and poor prognosis.^5^ Accurate biochemical assessment of HER2 with IHC and reflex ISH is paramount since initiation of anti-HER2 therapy improves oncologic outcomes. Historically, on the basis of these assays, HER2 IHC3+ and IHC2+/ISH+ patients have been combined into a single HER2+ entity. However, with a recent landmark trial establishing the new category of HER2-low disease, re-examination of HER2+ classification is warranted.^6^ The objective of this study was to explore whether HER2 IHC3+ and IHC2+/ISH+ patients undergoing NT anti-HER2 therapy, followed by surgery, exhibit distinct clinicopathologic features and/or treatment responses. We demonstrated that among the entire HER2+ patient cohort, unadjusted total pCR was 44.3%, similar to that seen prospectively.^16^ More important, IHC3+ status conferred a greater than threefold odds of pCR in adjusted analysis. The direct association between HER2 amplification and pathologic response was also observed in unadjusted subgroup analyses of HR+ and T1cN0 patients. Among IHC2+/ISH+ patients, pathologic response correlated with increased gene expression, together providing readily available clinical variables upon which to tailor NT management.

The central finding of this study is that HER2 IHC3+ status is independently associated with breast, axillary, and total pCR. To our knowledge, our study is the first to identify the impact of HER2+ IHC protein amplification on pathologic response rates using a large, contemporary US population database. Previous research examining outcomes between HER2 IHC3+ versus IHC2+/ISH+ patients treated in the adjuvant or metastatic setting found no clinical differences,^17,18^ with a paucity of studies evaluating pCR after NT anti-HER2 treatment, despite this being standard of care for the majority of cT2 cN+ HER2+ patients. Horimoto et al. reported a 45% versus 21% breast pCR rate in IHC3+ and IHC2+/ISH+ patients, but this difference was not statistically significant. Additional retrospective, mostly single-institution studies have demonstrated significantly improved pCR rates among IHC3+ patients, although each study employed non-standardized pCR definitions and/or small sample sizes.^12,19–22^ In one larger, single-institution study examining 531 consecutive HER2+ patients who received NT and surgery, pCR was 67% versus 17% for IHC3+ and IHC2+/ISH+.^22^ This parallels our study’s total pCR of 49.4% versus 16.3% for IHC3+ versus IHC2+/ISH+ patients, with the observed reduction in IHC3+ pCR likely stemming from the highly varied practice patterns of NCDB centers versus those of a single, large cancer center.

As previously mentioned, NT anti-HER2 therapy is standard of care for HER2+ patients with cT2+ or cN+ disease to downstage the breast and/or axilla with the eventual goal of surgical de-escalation. Yet, achieving pCR is a major treatment goal in its own right since this outcome is associated with prolonged event-free and overall survival.^23^ For this reason, the NCCN also advises consideration of NT for HER2+ cT1c breast cancers.^2^ Unfortunately, data to guide these treatment decisions are sparse. Published in 2016, the NeoSphere Trial established the superiority of neoadjuvant dual-agent pertuzumab and trastuzumab (P+T) to single-agent pertuzumab for achieving pCR, but the study did not include cT1 patients.^24^ Similarly, the 2019 KRISTINE trial, which compared NT trastuzumab emtansine (T-DM1) and pertuzumab with standard docetaxel, carboplatin, and P+T, also excluded HER2+ cT1 patients.^25^ Thus, a sub-aim of this study was to investigate pCR among HER2+ cT1c patients and determine whether HER2 amplification was related to pathologic response. We found that among cT1c cN0 patients, breast pCR rate was 46.2%, similar to the 43.3% pCR rate demonstrated by another NCDB study spanning an earlier timeframe.^26^ Consistent with our other analyses, breast pCR was significantly improved for cT1c HER2 IHC3+ compared with IHC2+/ISH+ patients (48.0% vs. 21.7%, *p* < 0.0001). Pathologic response further declined after stratifying by HR status, with a breast pCR of only 17.8% for the HR+ HER2+ IHC2+/ISH+ group. Given that breast pCR was low and on par with that quoted in the literature for HR+/HER2− disease, for which NT is not routinely recommended,^2,27^ cT1c HR+ HER2 IHC2+/ISH+ patients may represent a subgroup more appropriate for a surgery-first approach. On the other hand, NT may be more reasonable for cT1c HER2 IHC3+ patients, in whom higher response rates may justify the risk of toxicity and possible axillary overtreatment.

Our study is also novel because it evaluated pCR in the breast and nodal basins separately, as well as combined (i.e. *total* pCR), adding to the growing literature on discordant pathologic responses. We found that for all subgroups, rates of nodal pCR were superior to breast pCR, consistent with multiple previous publications.^28–30^ Although a more stringent pCR definition (ypT0/is ypN0) may serve as a prognostic endpoint,^15,23,31–33^ examining discrete pathologic responses in the breast and/or axilla is clinically relevant. For instance, residual disease in either the breast or axilla qualifies patients for escalation of adjuvant treatment to T-DM1, according to the KATHERINE trial.^34^ Additionally, an axillary pCR, based initially on intraoperative pathologic assessment, can obviate the need for completion lymphadenectomy, independent of breast pCR. In their phase II study, Weiss et al. found that HER2+ breast pCR consistently predicted ypN0, regardless of initial clinical nodal category. The authors suggest that in the future, nodal staging could be omitted following NT in highly selected HER2+ patients based on breast pCR, with prospective clinical trials currently underway to investigate the question of tailoring surgical de-escalation.^16^ Such examples highlight how clearer understanding of pCR in the breast and axilla individually may advance clinical management.

Phenotypic differences in therapeutic response by IHC status may be related to tumor heterogeneity*,* which refers to the diverse patterns of HER2 expression that lead to distinct subpopulations of cells within a single tumor. Although tumor heterogeneity is present in up to 34% of breast cancers, it is more common in HER2 equivocal patients, likely owing to the intermixing of HER2-amplified and non-amplified populations on the cell surface.^1^ Clinically, HER2 heterogeneity is associated with therapeutic resistance, poor prognosis, and worse clinical outcomes, including larger tumor size, higher histologic grade, greater frequency of lymph node metastases, and shorter survival.^1^ Using a heterogeneity definition of HER2+ in 5–50% of tumor cells, a recent phase II clinical trial uncovered a 10% rate of heterogeneity among HER2+ patients treated with six cycles of NT pertuzumab and T-DM1. Strikingly, there was poor treatment efficacy among heterogenous tumors, with pCR of 55% in non-heterogeneous tumors versus 0% in heterogenous tumors, 75% of which were IHC2+/ISH+.^35^ With rising interest in antibody drug conjugate therapy, tumor heterogeneity may become a relevant marker for selecting HER2+ patients who stand to gain greatest therapeutic benefit, but further studies are needed to integrate this parameter into practice.

Numerous additional covariates predicted breast, axilla, or total pCR. Socioeconomic and demographic factors, such as median income, insurance, and race, all proxies for health care access and therapy completion, were associated with pathologic response. Specifically, pCR was increased among patients with private insurance, which may protect patients from financial toxicity that can interfere with completing NT. In this study, Black race was also associated with decreased odds of nodal and total pCR. This finding aligns with previous research showing that Black women tend to present with distinct disease characteristics, receive lower doses of systemic therapy, and/or experience delays or early discontinuation of therapy, although further exploration of these disparities was beyond the scope of this study.^36,37^ For breast, axillary, and total pCR, higher grade and HR-negative tumors were independently associated with pCR, while greater T category was associated with decreased odds of breast pCR. Finally, although breast pCR rates were lower than nodal pCR rates in the overall cohort (48.7% vs. 67.3%), clinical N category was an independent predictor of breast, but not axillary, pCR. This discrepancy may reflect differences in the immune surveillance, tumor-infiltrating lymphocytes, therapy sensitivity, and/or HER2 amplification between the primary site and regional nodal basin, or disruption of the circulation to lymphatic tissue.^28,38^

This study has several limitations, in addition to its retrospective design. Although use of a large cancer database to answer population-level questions is beneficial, there are several drawbacks to NCDB data. Even after strict inclusion and exclusion criteria to refine the patient population, the very large study sample may overpower the results, suggesting statistically significant differences that are not clinically relevant. The NCDB also lacks granularity, including exact treatment regimens, dose reductions or delays, molecular subtypes, histologic data, and short- and long-term outcomes. Further multi-institutional or prospective studies are needed to better understand the impact of HER2 IHC status on pCR, as well as recurrence, progression, or event-free survival. Despite these shortcomings, our study demonstrates that HER2+ IHC status, data readily available from the pretreatment breast biopsy, independently predicts pCR prior to surgery, providing invaluable information for patient counseling and clinical management.

## Conclusion

This is the first large, population-based study to establish that HER2 IHC3+ and IHC2+/ISH+ breast cancers have distinct pathologic responses to neoadjuvant systemic therapy. These findings support the notion that IHC status may serve as a factor, along with HR and gene expression, to select which HER2-overexpressing patients derive the greatest benefit from neoadjuvant anti-HER2 treatment. More broadly, these data lend support for the continued reexamination of HER2 classification, as well as illustrate the importance of developing additional diagnostic tools to predict therapeutic response and guide clinical management.

## Supplementary Information

Below is the link to the electronic supplementary material.

Supplementary file1 (DOCX 33 KB)
