# Supplementary material for: Immunohistochemical Status Predicts Pathologic Complete Response to Neoadjuvant Therapy in HER2-Overexpressing Breast Cancers
Source: Ann Surg Oncol. 2024 Nov 9;32(2):931–43. doi: 10.1245/s10434-024-16470-8 (PMC11698782; doi:10.1245/s10434-024-16470-8)
Supplement: Supplementary file 1 — Supplementary file1 (DOCX 33 KB) [file 10434_2024_16470_MOESM1_ESM.docx]

**Supplemental Table 1. Demographics and clinicopathologic characteristics of clinical stage I-III invasive breast cancer patients with available HER2+ status stratified by HER2 subgroups.**

|  |  | **Overall** | **HER2 status** | | | **p** |
| --- | --- | --- | --- | --- | --- | --- |
|  |  |  | ***Negative**** | ***IHC2+/ISH+*** | ***IHC3+*** |  |
| **n** |  |  |  |  |  |  |
| **Age at diagnosis, years (Median (IQR))** | | 61 (51-70) | 62 (52-70) | 60 (50-69) | 56 (47-66) | <0.0001 |
| **Race/Ethnicity** | |  |  |  |  | <0.0001 |
|  | White | 680835 (81.2%) | 587659 (81.8%) | 20238 (78.6%) | 72938 (77.6%) |  |
|  | Black | 99432 (11.9%) | 83252 (11.6%) | 3592 (14.0%) | 12588 (13.4%) |  |
|  | Asian | 37537 (4.5%) | 30441 (4.2%) | 1276 (5.0%) | 5820 (6.2%) |  |
|  | Other/Unknown | 20321 (2.4%) | 17088 (2.4%) | 627 (2.4%) | 2606 (2.8%) |  |
| **Charlson/Deyo Score** | |  |  |  |  | <0.0001 |
|  | 0 | 692375 (82.6%) | 591383 (82.3%) | 21159 (82.2%) | 79833 (85.0%) |  |
|  | 1 | 106082 (12.7%) | 92116 (12.8%) | 3346 (13.0%) | 10620 (11.3%) |  |
|  | ≥2 | 39668 (4.7%) | 34941 (4.9%) | 1228 (4.8%) | 3499 (3.7%) |  |
| **Insurance Status** | |  |  |  |  | <0.0001 |
|  | Not Insured | 13597 (1.6%) | 10870 (1.5%) | 497 (1.9%) | 2230 (2.4%) |  |
|  | Private Insurance | 432052 (51.5%) | 362817 (50.5%) | 13774 (53.6%) | 55461 (59.0%) |  |
|  | Medicaid | 58927 (7.0%) | 47867 (6.7%) | 2081 (8.1%) | 8979 (9.6%) |  |
|  | Medicare | 315648 (37.7%) | 281738 (39.2%) | 8828 (34.2%) | 25082 (26.7%) |  |
|  | Other Government | 9430 (1.1%) | 7949 (1.1%) | 276 (1.1%) | 1205 (1.3%) |  |
|  | Unknown | 8471 (1.0%) | 7199 (1.0%) | 277 (1.1%) | 995 (1.1%) |  |
| **No High School Degree** | |  |  |  |  | <0.0001 |
|  | ≥ 17.6% | 129547 (15.5%) | 109014 (15.2%) | 4269 (16.6%) | 16264 (17.3%) |  |
|  | 10.9% - 17.5% | 180049 (21.5%) | 153955 (21.4%) | 5667 (22.0%) | 20427 (21.7%) |  |
|  | 6.3% - 10.8% | 210150 (25.1%) | 180941 (25.2%) | 6319 (24.6%) | 22890 (24.4%) |  |
|  | < 6.3% | 197885 (23.6%) | 171003 (23.8%) | 5803 (22.6%) | 21079 (22.4%) |  |
|  | Unknown | 120494 (14.4%) | 103527 (14.4%) | 3675 (14.3%) | 13292 (14.1%) |  |
| **Median Income** | |  |  |  |  | <0.0001 |
|  | < $40,227 | 103278 (12.3%) | 87667 (12.2%) | 3390 (13.2%) | 12221 (13.0%) |  |
|  | $40,227 - $50,353 | 140613 (16.8%) | 120400 (16.8%) | 4312 (16.8%) | 15901 (16.9%) |  |
|  | $50,354 - $63,332 | 167864 (20.0%) | 143968 (20.0%) | 5027 (19.5%) | 18869 (20.1%) |  |
|  | ≥ $63,333 | 304523 (36.3%) | 261721 (36.4%) | 9293 (36.1%) | 33509 (35.7%) |  |
|  | Unknown | 121847 (14.5%) | 104684 (14.6%) | 3711 (14.4%) | 13452 (14.3%) |  |
| **Institution Type** | |  |  |  |  | <0.0001 |
|  | Community Cancer Center | 56560 (6.7%) | 48724 (6.8%) | 1681 (6.5%) | 6155 (6.6%) |  |
|  | Comprehensive Community Cancer Program | 325872 (38.9%) | 282377 (39.3%) | 9611 (37.3%) | 33884 (36.1%) |  |
|  | Academic/Research Program | 247637 (29.5%) | 212552 (29.6%) | 7873 (30.6%) | 27212 (29.0%) |  |
|  | Integrated Network Cancer Program | 161134 (19.2%) | 140031 (19.5%) | 4655 (18.1%) | 16448 (17.5%) |  |
|  | Unknown | 46922 (5.6%) | 34756 (4.8%) | 1913 (7.4%) | 10253 (10.9%) |  |
| **Histology** | |  |  |  |  | <0.0001 |
|  | Ductal | 742455 (88.6%) | 628170 (87.4%) | 24244 (94.2%) | 90041 (95.8%) |  |
|  | Lobular | 84842 (10.1%) | 80812 (11.2%) | 1199 (4.7%) | 2831 (3.0%) |  |
|  | Mixed/Other | 10828 (1.3%) | 9458 (1.3%) | 290 (1.1%) | 1080 (1.1%) |  |
| **Estrogen Receptor** | |  |  |  |  | <0.0001 |
|  | Negative | 139872 (16.7%) | 103400 (14.4%) | 4680 (18.2%) | 31792 (33.8%) |  |
|  | Positive | 698253 (83.3%) | 615040 (85.6%) | 21053 (81.8%) | 62160 (66.2%) |  |
| **Progesterone Receptor** | |  |  |  |  | <0.0001 |
|  | Negative | 216648 (25.8%) | 162421 (22.6%) | 7436 (28.9%) | 46791 (49.8%) |  |
|  | Positive | 621477 (74.2%) | 556019 (77.4%) | 18297 (71.1%) | 47161 (50.2%) |  |
| **Tumor Grade** | |  |  |  |  | <0.0001 |
|  | Low | 185736 (22.2%) | 180834 (25.2%) | 1469 (5.7%) | 3433 (3.7%) |  |
|  | Intermediate | 376688 (44.9%) | 332490 (46.3%) | 10881 (42.3%) | 33317 (35.5%) |  |
|  | High | 253328 (30.2%) | 187542 (26.1%) | 12674 (49.3%) | 53112 (56.5%) |  |
|  | Undifferentiated | 537 (0.1%) | 411 (0.1%) | 20 (0.1) | 106 (0.1%) |  |
|  | Unknown/Missing | 21836 (2.6%) | 17163 (2.4%) | 689 (2.7%) | 3984 (4.2%) |  |
| **Lymphovascular Invasion** | |  |  |  |  | <0.0001 |
|  | Absent | 575567 (68.7%) | 505388 (70.3%) | 15404 (59.9%) | 54825 (58.4%) |  |
|  | Present | 132814 (15.8%) | 110170 (15.3%) | 5401 (21.0%) | 17243 (18.4%) |  |
|  | Unknown | 129368 (15.4%) | 102608 (14.3%) | 4923 (19.1%) | 21837 (23.2%) |  |
| **Clinical Tumor Stage** | |  |  |  |  | <0.0001 |
|  | cT1 | 532224 (63.5%) | 476994 (66.4%) | 12841 (49.9%) | 42389 (45.1%) |  |
|  | cT2 | 238431 (28.4%) | 191057 (26.6%) | 9945 (38.6%) | 37429 (39.8%) |  |
|  | cT3 | 45996 (5.5%) | 34830 (4.8%) | 1958 (7.6%) | 9208 (9.8%) |  |
|  | cT4 | 21474 (2.6%) | 15559 (2.2%) | 989 (3.8%) | 4926 (5.2%) |  |
| **Clinical Nodal Stage** | |  |  |  |  | <0.0001 |
|  | cN0 | 695884 (83.0%) | 613972 (85.5%) | 19144 (74.4%) | 62768 (66.8%) |  |
|  | cN1 | 118582 (14.1%) | 87521 (12.2%) | 5469 (21.3%) | 25592 (27.2%) |  |
|  | cN2 | 14101 (1.7%) | 10255 (1.4%) | 668 (2.6%) | 3178 (3.4%) |  |
|  | cN3 | 9558 (1.1%) | 6692 (0.9%) | 452 (1.8%) | 2414 (2.6%) |  |
| **Anti-HER2 therapy** | | 98757 (11.8%) | 8996 (1.3%) | 18604 (72.3%) | 71157 (75.7%) | <0.0001 |
| **Breast Surgery** | |  |  |  |  | <0.0001 |
|  | None | 26533 (3.2%) | 21550 (3.0%) | 993 (3.9%) | 3990 (4.2%) |  |
|  | Partial Mastectomy | 513152 (61.2%) | 454102 (63.2%) | 13768 (53.5%) | 45282 (48.2%) |  |
|  | Mastectomy | 297175 (35.5%) | 241703 (33.6%) | 10943 (42.5%) | 44529 (47.4%) |  |
|  | Unknown | 1265 (0.2%) | 1085 (0.2%) | 29 (0.1%) | 151 (0.2%) |  |
| **Axillary Surgery** | |  |  |  |  | <0.0001 |
|  | None | 61912 (7.4%) | 53877 (7.5%) | 1692 (6.6%) | 6343 (6.8%) |  |
|  | SLNB alone | 542916 (64.8%) | 472636 (65.8%) | 15361 (59.7%) | 54919 (58.5%) |  |
|  | SNLB then ALND | 126384 (15.1%) | 106837 (14.9%) | 4088 (17.7%) | 15002 (16.0%) |  |
|  | ALND alone | 105428 (12.6%) | 83922 (11.7%) | 4163 (16.0%) | 17418 (18.5%) |  |
|  | Unknown | 1485 (0.2%) | 1168 (0.2%) | 47 (0.2%) | 270 (0.3%) |  |

Axillary lymph node dissection, ALND; centromere enumerator probe 17, CEP17; estrogen receptor, ER; human epidermal growth factor receptor 2, HER2; immunohistochemistry, IHC; interquartile range, IQR; progesterone receptor, PR; sentinel lymph node biopsy, SLNB

* HER2 negative is defined as IHC 0, IHC 1+ or IHC 2+/ISH-.

**Supplemental Table 2. Distribution of ISH ratios among HER2+ IHC2+ patients achieving breast, nodal, and total pathologic responses.**

|  |  | **Breast pCR** | | | | | **Nodal pCR*** | | | | | **Total pCR **** | | | | |
| --- | --- | --- | --- | --- | --- | --- | --- | --- | --- | --- | --- | --- | --- | --- | --- | --- |
|  |  | **All** | **No** | | **Yes** | | **All** | **No** | | **Yes** | | **All** | **No** | | **Yes**** | |
|  |  | **N** | **No** | **%** | **No** | **%** | **N** | **No** | **%** | **No** | **%** | **N** | **No** | **%** | **No** | **%** |
|  |  | 5848 | 4592 | 78.5 | 1256 | 21.5 | 2711 | 1744 | 64.3 | 967 | 35.7 | 2711 | 1596 | 58.9 | 414 | 15.3 |
| **HER2 ISH Ratio** | |  |  |  |  |  |  |  |  |  |  |  |  |  |  |  |
|  | <2 | 738 | 609 | 82.5 | 129 | 17.5 | 332 | 231 | 69.6 | 101 | 30.4 | 332 | 218 | 65.7 | 37 | 11.1 |
|  | ≥2 | 5110 | 3983 | 77.9 | 1127 | 22.1 | 2379 | 1513 | 63.6 | 866 | 36.4 | 2379 | 1378 | 57.9 | 377 | 15.8 |
| **ISH Ratio Categories** | |  |  |  |  |  |  |  |  |  |  |  |  |  |  |  |
|  | 0.20 - 1.99 | 738 | 609 | 82.5 | 129 | 17.5 | 332 | 231 | 69.6 | 101 | 30.4 | 332 | 218 | 65.7 | 37 | 11.1 |
|  | 2.00 - 2.25 | 1284 | 1064 | 82.9 | 220 | 17.1 | 654 | 467 | 71.4 | 187 | 28.6 | 654 | 436 | 66.7 | 68 | 10.4 |
|  | 2.26 - 2.50 | 923 | 755 | 81.8 | 168 | 18.2 | 415 | 276 | 66.5 | 139 | 33.5 | 415 | 256 | 61.7 | 48 | 11.6 |
|  | 2.51 - 3.00 | 901 | 713 | 79.1 | 188 | 20.9 | 401 | 261 | 65.1 | 140 | 34.9 | 401 | 233 | 58.1 | 59 | 14.7 |
|  | 3.01 - 4.00 | 799 | 610 | 76.3 | 189 | 23.7 | 367 | 221 | 60.2 | 146 | 39.8 | 367 | 201 | 54.8 | 65 | 17.7 |
|  | 4.01 - 6.00 | 725 | 520 | 71.7 | 205 | 28.3 | 327 | 182 | 55.7 | 145 | 44.3 | 327 | 159 | 48.6 | 78 | 23.9 |
|  | 6.01 - 96.1 | 478 | 321 | 67.2 | 157 | 32.8 | 215 | 106 | 49.3 | 109 | 50.7 | 215 | 93 | 43.3 | 59 | 27.4 |

Human epidermal growth factor receptor 2, HER2; in situ hybridization, ISH; pathologic complete response, pCR

*Excludes cN0 patients

** Patients with breast or nodal pCR only are included in 'no Total pCR'; excludes cN0 patients
